# The Anti-Oxidation and Mechanism of Essential Oil of *Paederia scandens* in the NAFLD Model of Chicken

**DOI:** 10.3390/ani9100850

**Published:** 2019-10-22

**Authors:** Qiang Wu, Huaqiao Tang, Hongbin Wang

**Affiliations:** 1 College of Animal Medicine, Northeast Agricultural University, Harbin 150030, China; wuqiangfirst@163.com; 2 Agricultural College, Tongren Polytechnic College, Tongren 554300, China; 3 Department Pharmacy, Sichuan Agricultural University, Chengdu 611130, China; turtletang@163.com

**Keywords:** oxidative stress, essential oil of *Paederia scandens*, non-alcoholic fatty liver disease, HSP7C, chicken

## Abstract

**Simple Summary:**

The essential oil of *Paederia scandens* can remedy non-alcoholic fatty liver disease of chicken, but the mechanisms remain unclear. In this study, proteomics technology was used to declare the anti-non-alcoholic fatty liver disease mechanism of *Paederia scandens* essential oil. The results show that the essential oil of *Paederia scandens* significant decreased the oxidative stress of non-alcoholic fatty liver disease in chicken, which was mainly due to the center regulation protein of HSP7C being significantly inhibited.

**Abstract:**

The aim of the study is to determine the underlying pathogenic mechanisms of oxidative stress and detect the anti-oxidative target of essential oil of *Paederia scandens* in non-alcoholic fatty liver disease (NAFLD). Chicken NAFLD was modeled by feeding with a high-capacity diet and *Paederia scandens* essential oil was used to treat the disease. The levels of hepatic reactive oxygen species (ROS), malondialdehyde (MDA), superoxide dismutase (SOD), and the differential proteins and network of protein–protein interactions were investigated in model and drug-treated groups. The results showed that essential oil of *Paederia scandens* down regulated the hepatic ROS and MDA level significantly (*p* < 0.05 and 0.01, respectively). The heat shock cognate 71 kDa protein (HSP7C) was down regulated significantly, which was in the center of the network and interacted with 22 other proteins. The results showed that oxidative stress played an important role in the pathogenesis of chicken NAFLD. The essential oil of *Paederia scandens* showed good anti-oxidation activity by down regulating the HSP7C protein, which can be used as a potential therapeutic target in chicken NAFLD.

## 1. Introduction

Non-alcoholic fatty liver disease (NAFLD) is the most common worldwide nutritional metabolic disease in the general population due to lifestyle changes, such as increased consumption of high-fat food and lack of exercise, which are also the main reasons for chronic diseases, including dyslipidemia, chronic kidney disease, obesity, and hyperglycemia [1,2]. Chickens are also prone to burst fatty liver disease due to factors such as nutrition, hormones, genetics, and environment. Chicken NAFLD affects the normal function of the liver, and even causes liver cell rupture, eventually leading to intrahepatic hemorrhage and death, which is an important reason for the lost in the poultry industry [3]. The estimated prevalence of chicken NAFLD is around 5%, but occasional outbreaks occur up to 20% and cause huge economic losses [4].

There is no sufficient explanation of the mechanisms which are associated with the development of NAFLD, including progression to other nosologic units. It is hypothesized that it depends on genetic and environmental factors, and eventual progression is the result of bilateral interactions [5]. The most widely accepted pathogenesis of NAFLD is the “two hits” hypotheses [6,7], in which lipid accumulation is considered as the first hit and increased oxidative stress is proposed to be the second hit. Lipid oxidation generates toxic metabolites including ROS and MDA, which can damage many cellular components such as DNA, protein, lipid, and mitochondria [8,9]. Meanwhile, ROS drives peroxidation of the accumulated hepatic lipids directly [10], which increases the formation of lipid peroxidation products, such as MDA, which is major aldehydic metabolites of lipid peroxidation and has been used to reflect lipid peroxidation [11] and stimulates further production of ROS [9].

Some studies have shown that oxidative stress may be the most critical pathogenesis of NAFLD leading to disease progression, and there can be an effective preventive and/or treatment strategy against chicken NAFLD by hepatoprotective effect or anti-oxidant activity. The recent study showed that essential oil of *Paederia scandens* has the activity of hepatoprotective and anti-oxidant effect [12]. However, the targets of hepatoprotective effects and anti-oxidant activity of essential oil of *Paederia scandens* are still unclear. This study was carried out on the intervention of a high-capacity diet and essential oil of *Paederia scandens*, which aimed to determine the underlying pathogenic mechanisms and to obtain the hepatoprotective target of this essential oil in chicken NAFLD.

## 2. Materials and Methods

### 2.1. Materials and Extraction

Plant materials were harvested from Guizhou (27.7183° N, 109.192° E, southwest China), in June 2015. All solvents and reagents were analytical grade. The harvested samples were washed with tap water and dried at 30 °C for 6 days by oven. A sample of 150 g crushed *Paederia scandens* was subjected to extraction by hydro distillation for 3 h in 500 mL distilled water using a clevenger type apparatus. The extracted oil was recovered and stored at 4 °C, and its extraction yield was calculated as the ratio of the weight of oil to the weight of fruits using the following equation:

% yield of oil = (weight of oil/weight of dried materials) × 100%(1)

### 2.2. High Performance Liquid Chromatography (HPLC) Analysis

The main ingredients of linalool, L-α-terpineol, dextro-α-terpineol, methyl salicylate, camphor, borneol, eugenol, and isoeugenol were chosen as the standards to determine the constituent of the essential oil of *Paederia scandens*. The standards and extraction were analyzed by Agilent 1260 high performance liquid chromatography (HPLC) equipped with an Agilent Zorbax Eclipse XDB-C8 column (4.6 mm × 250 mm, 5 μm) (Agilent Technologies, Santa Clara, CA, USA). The test condition was acetonitrile–water (55:45); flow rate 1.0 mL/min; injection volume 10 μL; wavelength 230 nm; column temperature 30 °C.

### 2.3. Animals and Experimental Procedure

Seventy-five, one-day-old, healthy Ross 305 chicks (33−40 g) were selected from the breeding chicken farm of Sichuan Wenjiang China Tai Livestock Co., Ltd. (Chengdu, China). All chickens were housed in wood cages under the recommended environment. Chicks were brooded at 33 °C during the first week; the brooding temperature was reduced 3 °C/week to approximately 24 °C by week four of age. Light was provided continually using incandescent lamps. The environmental humidity was controlled at 60−65%.

The chickens were randomly divided into control group, model group, and drug treated group, 25 individuals per group. The control group was given a normal diet, and model and drug-treated groups were given a high-capacity diet. Cooked pigs’ oil was the main calorie sources of the high-capacity diet. The drug group chickens were treated with essential oil of *Paederia scandens* by nasal drops, 2 mg/kg per day continuously for 4 weeks. The dose of *Paederia scandens* essential oil was calculated by previous experiments, and 2 mg/kg was the safe and effective dose to treat NAFLD in chicken [12]. Drug treated chickens were given the same diet as the model group chickens. The normal diet and high-capacity diet formulations are shown in Table 1. All chickens were given free access to laboratory feed and water for 4 weeks, and the chickens had no access to food for 12 h before they were anesthetized and sacrificed. Five chickens were randomly selected and killed weekly in each group, and the livers were excised and removed promptly for further analysis. All the animal tests were in accordance with the Administration of Affairs Concerning Experimental Animals of the State Council of the People’s Republic of China. The experiment protocol was approved by the Committee on Experimental Animal Management of Tongren Polytechnic college in Guizhou (with protocol No. 2016051407).

### 2.4. Oxidative Stress Assays

A small part of fresh liver was homogenized to prepare 10% tissue homogenate by adding 0.9% saline on ice in each group. The homogenate was centrifuged at 1500× *g* for 10 min at 0 °C. The supernatant was harvested and adjusted to 1% tissue homogenate by adding 0.9% saline. The levels of MDA, SOD and ROS were measured by assays kits from the NanJing Jiancheng bio-engineering research institute (NanJing, China).

### 2.5. Protein Separation and Identification

The liver (1.0 g) was suspended in 10 mL buffer consisting of 50 mM Tris and 1.0 mM phenylmethylsulphonyl fluoride for preparation of the total protein extract. The suspension was homogenized with the homogenizer for 1 min, sonicated for 30 s, and centrifuged at 14,000× *g* for 15 min in the buffer. The homogenate was poured into a glass beaker, which was placed on ice, and the homogenate was stirred gently for 30 min at 4 °C. Cell debris and other particulate matter were removed from the homogenate by centrifugation at 14,000× *g* for 20 min at 4 °C. After filtering the supernatant, the cells were washed by adding 10 mL phosphate buffer saline to the centrifuge tube. The cell pellet was resuspended by pipetting the mixture up and down. The cells were centrifuged again at 4800× *g* for 5 min at 4 °C. The supernatant was discarded without disturbing the pellet. A pipette was used to transfer the extraction buffer into the tube and vortex the tube briefly (10 s) to resuspend the pellet. Samples of 1.0 mg protein were applied on immobilized pH 3−10 nonlinear strips. Each sample was analyzed in triplicate. Focusing started at 200 V at 3 V/min and was kept constant for a further 24 h. The second-dimensional separation was performed in 12% SDS–polyacrylamide gels. The gels (18 × 18 × 0.15 cm) were run at 40 mA per gel. After protein fixation for 12 h in 40% methanol containing 5% phosphoric acid, the gels were stained with Coomassie blue R250 (0.5 g/L) for 24 h. The gels were scanned as tiff files and analyzed by PDQuest 8.01 (Bio-Rad, Berkeley, CA, USA). The significantly different pots were selected for MALDI-TOF/MS analysis. These proteins were identified by GPM-XE software (The Global Proteome Machine Organisation, USA, www.thegpm.org) and the network of protein–protein interactions was analyzed by Cytoscape v2.6.3 (U.S. National Institute of General Medical Sciences, USA, http://www.cytoscape.org).

### 2.6. Statistical Analysis

The statistical analyses were performed using the SPSS 19.0 software package (ICM, Armonk, NY, USA) and the data were presented as means and standard deviations (means ± SD), and group comparison was done by independent-sample *t*-tests.

## 3. Results

### 3.1. Paederia scandens Essential Oil Chemical composition

**Figure 1:**
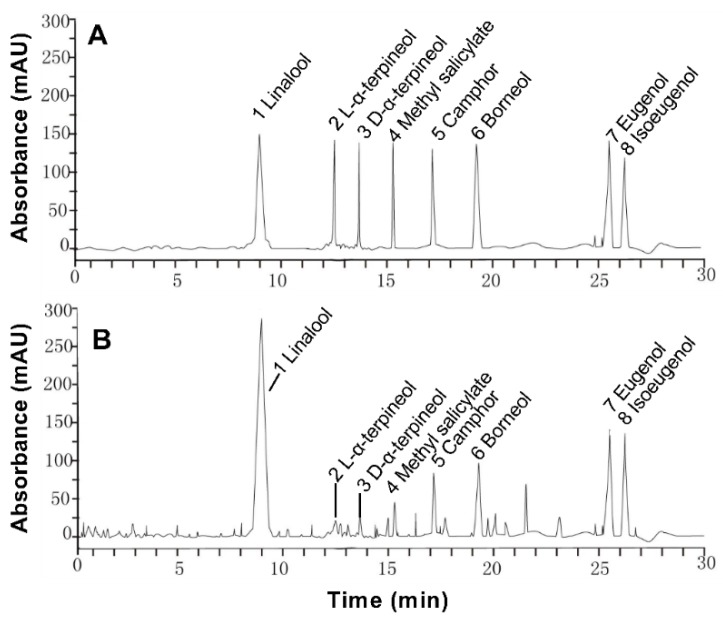
High performance liquid chromatogram of 100 μg/L mixed standard solution (**A**) and 500 μg/L in *Paederia scandens* essential oil (**B**).

The extraction rate of essential oil was about 0.5% in *Paederia scandens*. Eight chemical ingredients were tested by HPLC, and the contents were shown in Table 2. The main ingredients were linalool and borneol, the contents were 261.142 and 118.784 mg/mL in the oil, the results showed in Table 2. These eight components account for 54.14% of essential oil. The HPLC chromatogram is shown in Figure 1.

### 3.2. Clinical Symptoms and Relative Weight of the Liver

The clinical manifestations were weight gain, unstable standing, mouth breathing, lethargy, unilateral lying, increased body temperature, and feces rot in most of the chickens treated with high-calorie diets. Even a small number of chickens died. There was no chicken death in the drug treatment group, no obvious clinical features, and the chickens grew well.

In the model group, most chickens showed a large amount of yellow–brown fat deposition in the abdominal cavity and mesentery. The liver was swollen (data shown in Table 3), deep yellow, soft, and brittle. Some livers lost their original shape. In the drug treatment group, the hepatic hemorrhage band was reduced or even disappeared, and some showed small bleeding spots. The overall liver appearance was similar to that of the normal group.

### 3.3. Hepatic ROS, MDA, and Superoxidase Dismutase (SOD) Levels

The results showed that hepatic ROS levels were significantly higher in the model and drug groups than in the control group during the whole experiment (*p* < 0.01 or *p* < 0.05), and hepatic ROS levels were lower in the drug group than in the model group in the early stage. Hepatic MDA levels were significantly higher in the model group than in the drug group and control group from 2 weeks to 4 weeks (*p* < 0.01 or *p* < 0.05). Hepatic SOD activity was significantly lower in the model group and drug group than in the control group from 3 weeks (*p* < 0.01 or *p* < 0.05). The essential oil of *Paederia scandens* significantly down regulated the hepatic ROS and MDA levels in NAFLD chicken. The results are listed in Table 4.

### 3.4. Two-Dimensional (2D) Gel Electrophoresis

**Figure 2:**
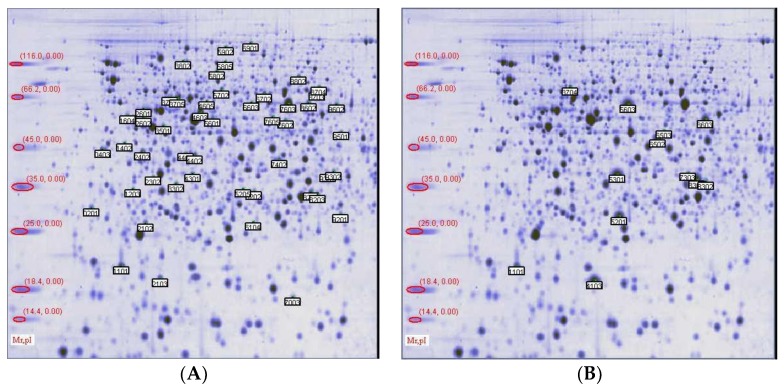
Two-dimension gel electrophoresis of proteins from liver extract stained with Coomassie Brilliant Blue. pH 3−10 nonlinear first dimension, and 12% SDS–PAGE second dimension. (**A**) shows that there were 73 differential spots in the drug-treated group compared with model group; (**B**) shows that there were 22 differential spots in the drug-treated group compared with control group. Differentially expressed proteins are marked in each spot sustained an individual identification number (SSP).

The total proteins were analyzed by 2D electrophoresis. The samples were analyzed on broad pH range immobilized pH gradient (IPG) strips and the spots were visualized following staining with Coomassie brilliant blue. The results showed a representative analysis of total liver proteins separated on a broad pH range 3−10 2D gel. On each gel, 1.0 mg of total protein amount was applied. The analysis of two-dimensional maps in PDQuest showed that there were about 400 spots detected by Commassie Brilliant Blue-stained in each group. There were 73 markedly differential spots in B gel compared with group A, and there were 22 markedly differential spots in C gel compared with B gel. These differential spots are shown on Figure 2.

### 3.5. Protein Separation and Identification

In total, 22 differential proteins were identified in C gel, eight of which were up-regulation, such as heat shock 70 kDa protein 5, heat shock 70 kDa protein 8, long-chain specific acyl-CoA dehydrogenase, etc. There were four proteins with the function of lipid metabolism and fatty acid beta-oxidation, such as 3-alpha-hydroxysteroid dehydrogenase, 3-hydroxy-3-methylglutaryl-Coenzyme A synthase 2, Carbonic anhydrase 3, and Catalase. Additionally, there were four proteins with the function of electron transport and cell communication, such as electron transfer flavoprotein subunit alpha, ATP synthase subunit d, Regucalcin, and Guanine nucleotide-binding protein subunit beta-2-like 1. The results are listed in Table 5.

### 3.6. Analysis of the Network of Protein–Protein Interactions

**Figure 3:**
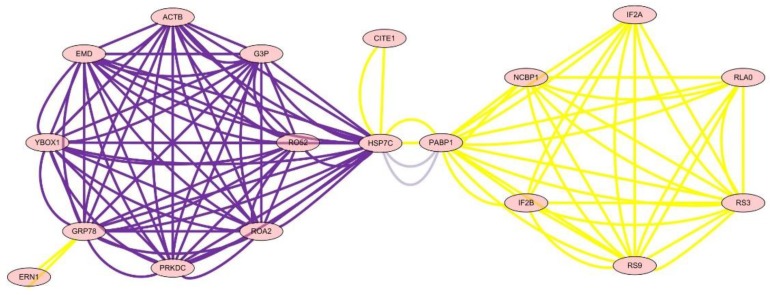
The network of differential protein expression.

The analysis of the network of protein–protein interactions was done by Cytoscape v2.6.3. The results showed that HSP7C, heat shock cognate 71 kDa protein, was in the center of the network and interaction among 22 other proteins, which showed that essential oil of *Paederia scandens* had the activity of hepatoprotective effect and anti-oxidant by up-regulation of HSP7C expression and affected other proteins by interaction. The results are listed in Table 6 and Figure 3.

## 4. Discussion

Oxidative stress can be described as a condition resulting from an uncontrolled increase in ROS and MDA or an insufficiency in the anti-oxidant system under certain pathological states [13]. The balance of oxidation and the anti-oxidation system plays an essential role in the progression and pathogenesis of NAFLD [14]. In the present study, we established the high-capacity induced NAFLD model in chicken and explored the degree and mechanisms of oxidative stress through detecting the hepatic ROS, MDA, and SOD. The key proteins were screened and the anti-oxidative target of essential oil of *Paederia scandens* was determined in chicken NAFLD.

The results showed that hepatic ROS and MDA levels were significantly higher in the model group than other groups, but SOD activity was significantly lower at the same time, which indicated that oxidative stress occurred and played a significant role in liver injury and disease progression in chicken NAFLD. Recent studies have shown that high levels of lipid accumulation exceeded the storage capacity of liver in NAFLD [15], which led to lipid peroxidation, thereby causing production of MDA and ROS with important toxic effects [16]. Excessive production of ROS and MDA may eventually overwhelm anti-oxidant defenses and generate highly toxic lipid peroxides [17]. The activity of SOD, sensitive to inactivation by ROS and MDA, was decreased in the NAFLD chicken [18,19]. Furthermore, to prevent oxidative stress, there was a continuous balance between intrahepatic antioxidants and oxidative stress, but an imbalance between the increased ROS and MDA level, and the decreased SOD activity occurred in the model group, which contributed to the pathogenesis of chicken NAFLD [20,21]. Meanwhile, hepatic ROS and MDA levels were significantly lower in the drug group than in the model group, which demonstrated the anti-oxidative activity of essential oil of *Paederia scandens*.

The differential proteins were analyzed by two-dimensional (2D) gel electrophoresis and mass spectrometry in chicken NAFLD and drug treated groups, which indicated that chicken NAFLD followed many differential protein expressions, and essential oil of *Paederia scandens* can up or down regulate some proteins, which is related to the hepatic ROS and MDA level regulation by the network of protein–protein interactions. There were 22 differential proteins in the drug-treated group compared with the model group. Of these, there were four proteins with the function of lipid metabolism and fatty acid beta-oxidation, such as 3-alpha-hydroxysteroid dehydrogenase, 3-hydroxy-3-methylglutaryl-Coenzyme A synthase 2, Carbonic anhydrase 3, and Catalase. Additionally, there were four proteins with the function of electron transport and cell communication, such as electron transfer flavoprotein subunit alpha, ATP synthase subunit d, Regucalcin, and Guanine nucleotide-binding protein subunit beta-2-like 1. Eight proteins were up-regulated, such as heat shock 70 kDa protein 5 (glucose-regulated protein, 78 kDa), heat shock 70 kDa protein 8, Long-chain specific acyl-CoA dehydrogenase, Beta-actin, and 3-hydroxy-3-methylglutaryl-Coenzyme A synthase 2.

To define the mechanism of anti-oxidation and further determine the targets of essential oil of *Paederia scandens* in treating chicken NAFLD, the protein network analysis results showed that HSP7C, heat shock cognate 71 kDa protein, was in the center of the network and interaction among the other 22 proteins, which indicated that HSP7C may be a core target for essential oil of *Paederia scandens* against chicken NAFLD. Some recent studies showed that HSP7C is a member of the heat shock protein 70 (HSP70) family and is involved in the folding and assembly of proteins in the endoplasmic reticulum (ER). As this protein interacts with many ER proteins, it may play a key role in monitoring protein transport through the cell [22,23]. Above all, essential oil of *Paederia scandens* has a hepatoprotective effect and anti-oxidant activity by up-regulation of HSP7C expression, which interacts with other proteins; similar results were reported by Attia et al. [24].

## 5. Conclusions

In conclusion, excessive production of ROS and MDA and decreased SOD activity overwhelmed antioxidant defenses and further generated highly toxic lipid peroxides, which induced the formation of oxidative stress in chicken NAFLD. The essential oil of *Paederia scandens* has a hepatoprotective effect and anti-oxidant activity by up-regulation of HSP7C protein expression, which may be a potential therapeutic target in treating NAFLD chicken.

## Figures and Tables

**Table 1:** Composition and nutrient levels of the diets.

Ingredients (%)	High-Capacity Diets	Normal Diets
Corn	53.00	62.48
Soybean meal	-	-
Fish meal	1.00	3.00
Fried meal	29.30	28.10
Rapeseeds	-	2.20
Wheat bran	-	-
Cooked pigs’ oil	12.95	-
Additives	0.75	0.75
Lys	0.12	0.09
Met	0.12	0.18
CaHPO_4_	1.11	1.22
Limestone meal	1.52	1.82
Salt	0.13	0.16
ME (MJ/kg)	16.07	13.39
CP (%)	16.00	18.40
Ca	0.90	1.00
AP	0.35	0.45
Lys	1.00	1.10
Met	0.38	0.50

The additives contains 0.50% of microelements, per kg dietary contains Fe 80 mg, Zn 40 mg, Cu 8 mg, Mn 60 mg, I 0.35 mg, Se 0.15 mg, 0.20% of choline, Va 1500 IU, VD_3_ 200 IU, VE 10 IU, VK_3_ 0.05 mg, VB_1_ 1.80 mg, VB_2_ 3.60 mg, VB_12_ 0.01 mg, VB_7_ 0.15 mg, VB_9_ 0.55 mg, VB_5_ 35 mg, VB_3_ 10 mg, VB_6_ 3.50 mg.

**Table 2:** Contents of main components of essential oil in *Paederia scandens*.

No.	Ingredients	Raw Sample (mg/mL)
1	Linalool	261.142
2	L-α-terpineol	10.126
3	D-α-terpineol	11.233
4	Methyl salicylate	78.902
5	Camphor	15.234
6	Borneol	118.784
7	Eugenol	24.634
8	Isoeugenol	21.346

**Table 3:** The change of liver index (%) in broilers.

Time (Week)	Control Group	Model Group	Drug Group
2	2.20 ± 0.15	3.02 ± 0.18 **	3.02 ± 0.18 **
3	2.22 ± 0.21	3.15 ± 0.11 **	2.83 ± 0.21 **
4	2.22 ± 0.22	3.05 ± 0.30 **	2.84 ± 0.17

** means *p* < 0.01 vs. control group.

**Table 4:** The level of hepatic ROS, MDA, and SOD.

Item	Week	Control Group	Model Group	Drug Group
ROS (U/mg protein)	1	88.56 ± 1.44	113.34 ± 4.06 **	97.64 ± 1.62 *
	2	88.56 ± 2.20	106.66 ± 4.48 **	98.33 ± 1.39 *
	3	93.60 ± 3.55	107.49 ± 7.96 **	105.01 ± 6.20 *
	4	100.69 ± 6.33	125.00 ± 5.86 **	109.08 ± 4.46
MDA (nmol/mg protein)	1	6.57 ± 1.46	10.60 ± 1.34 **	9.04 ± 1.27 **
	2	8.32 ± 1.63	10.51 ± 1.29 **	9.47 ± 1.56 **
	3	8.82 ± 1.26	13.47 ± 1.66 **	10.42 ± 1.79 **
	4	9.57 ± 1.18	13.46 ± 1.42 **	9.94 ± 1.03
SOD (U/mg protein)	1	498.96 ± 35.83	461.32 ± 37.49	436.11 ± 43.69 *
	2	536.60 ± 31.67	501.19 ± 27.13	476.48 ± 26.99 **
	3	564.89 ± 26.22	492.36 ± 30.23 **	425.99 ± 26.58 **
	4	523.83 ± 29.05	443.22 ± 33.35 **	417.21 ± 30.79 **

* *p* < 0.05 and ** *p* < 0.01 vs. control group.

**Table 5:** Different proteins expression in chicken liver.

SSP	Protein	Gene	MW/PI	Trend
1101	Uncharacterized protein	-	-	-
1203	Regucalcin	*Rgn*	33/5.3	↓
3704	Heat shock 70 kDa protein 5 (glucose-regulated protein, 78 kDa)	*Hspa5*	72/5.1	↑
4103	ATP synthase subunit d	*Atp5h*	19/6.2	↓
4301	Guanine nucleotide-binding protein G(I)/G(S)/G(T) subunit beta 1	*Gnb1*	35/7.6	↓
4401	S-adenosylmethionine synthase isoform type-1	*MAT1A*	44/5.6	↓
4402	Fumarylacetoacetase	*Fah*	46/6.7	↓
5201	Carbonic anhydrase 3	*Car3*	29/6.9	↓
5301	3-alpha-hydroxysteroid dehydrogenase	*Akr1c2*	37/6.7	↑
5603	3-hydroxy-3-methylglutaryl-Coenzyme A synthase 2	*Hmgcs2*	57/8.9	↑
5702	Catalase	*Cat*	60/7.1	↑
6104	Glutathione S-transferase Mu 1	*Gstm1*	26/8.3	↓
6502	Arginosuccinate synthase 1	*Ass1*	46/7.6	↓
6503	Long-chain specific acyl-CoA dehydrogenase	*Acadl*	48/7.6	↑
6702	Heat shock 70 kDa protein 8	*Hspa8*	71/5.4	↑
6901	Uncharacterized protein	*-*	-	-
7003	Beta-actin	*actb*	15/5.7	↑
7303	Uncharacterized protein	*Ote*	39/8.9	
8301	Electron transfer flavoprotein subunit alpha	*Etfa*	35/8.6	↓
8302	Glutathione S-transferase Mu 2	*Gstm2*	28/8.2	↓
9301	Glyceraldehyde-3-phosphate dehydrogenase-like	*Gapdh*	36/8.4	↓
9501	Betaine-homocysteine S-methyltransferase 1	*Bhmt*	45/8.0	↓
8603	Uncharacterized protein	-	-	-

The spots representing total proteins were analyzed by PDQuest 8.01 software and identified by mass spectrometry and GPM-XE software. “↓” means the proteins were down regulated; “↑” means the proteins were up-regulated. SSP means each spot sustained an individual identification number. MW/PI means the ratio of molecular weight and isoelectric point.

**Table 6:** The analysis of the network among differential protein expression.

ID	Degree	Label	Gene	Name
146490	22	HSP7C	*HSPA8*	Heat shock cognate 71 kDa protein
145948	18	GRP78	*HSPA5*	78 kDa glucose-regulated protein
93344	16	PABP1	*PABPC1*	Emerin
71781	16	YBOX1	*YBX1*	Nuclease-sensitive element-binding protein 1
57072	16	PRKDC	*PRKDC*	Heterogeneous nuclear ribonucleoproteins A2/B1
51704	16	ACTB	*ACTB*	Polyadenylate-binding protein 1
50923	16	RO52	*TRIM21*	E3 ubiquitin-protein ligase TRIM21
50095	16	ROA2	*HNRNPA2B1*	DNA-dependent protein kinase catalytic subunit
15490	16	EMD	*EMD*	Actin, cytoplasmic 1
150257	16	G3P	*GAPDH*	Glyceraldehyde-3-phosphate dehydrogenase
89014	12	RS3	*RPS3*	40S ribosomal protein S9
88619	12	RS9	*RPS9*	40S ribosomal protein S3
5116	8	IF2B	*EIF2S2*	Eukaryotic translation initiation factor 2 subunit 2
50028	8	RLA0	*RPLP0*	60S acidic ribosomal protein P0
4817	8	IF2A	*EIF2S1*	Eukaryotic translation initiation factor 2 subunit 1
117387	8	NCBP1	*NCBP1*	Nuclear cap-binding protein subunit 1
95707	2	CITE1	*CITED1*	Cbp/p300-interacting trans-activator 1
15584	2	ERN1	*ERN1*	Serine/threonine-protein kinase/endoribonuclease IRE1
